# Lasing in Bose-Fermi mixtures

**DOI:** 10.1038/srep20091

**Published:** 2016-01-29

**Authors:** Vladimir P. Kochereshko, Mikhail V. Durnev, Lucien Besombes, Henri Mariette, Victor F. Sapega, Alexis Askitopoulos, Ivan G. Savenko, Timothy C. H. Liew, Ivan A. Shelykh, Alexey V. Platonov, Simeon I. Tsintzos, Z. Hatzopoulos, Pavlos G. Savvidis, Vladimir K. Kalevich, Mikhail M. Afanasiev, Vladimir A. Lukoshkin, Christian Schneider, Matthias Amthor, Christian Metzger, Martin Kamp, Sven Hoefling, Pavlos Lagoudakis, Alexey Kavokin

**Affiliations:** 1 Spin Optics Laboratory, Saint-Petersburg State University, 1, Ulianovskaya, 198504, St-Petersburg, Russia; 2 Ioffe Physical-Technical Institute, Russian Academy of Sciences, 26, Politechnicheskaya, 194021, St-Petersburg, Russia; 3 Institut Néel, CNRS/UJF 25, avenue des Martyrs - BP 166, Fr-38042 Grenoble Cedex 9, France; 4 Faculty of Physical Sciences and Engineering, University of Southampton, Highfield, Southampton, SO171BJ, UK; 5 Science Institute, University of Iceland, Dunhagi-3, IS-107, Reykjavik, Iceland; 6 Department of Applied Physics/COMP, Aalto University, PO Box 14100, 00076 Aalto, Finland; 7 Division of Physics and Applied Physics, Nanyang Technological University, 637371, Singapore; 8 Department of Materials Science & Technology, University of Crete, Greece; 9 IESL-FORTH, P.O. Box 1527, 71110 Heraklion, Crete, Greece; 10 Technische Physik and Wilhelm-Conrad-Röntgen-Research Center for Complex Material Systems, Universität Würzburg, D-97074 Würzburg, Am Hubland, Germany; 11 SUPA, School of Physics and Astronomy, University of St Andrews, St Andrews, KY16 9SS, United Kingdom

## Abstract

Light amplification by stimulated emission of radiation, well-known for revolutionising photonic science, has been realised primarily in fermionic systems including widely applied diode lasers. The prerequisite for fermionic lasing is the inversion of electronic population, which governs the lasing threshold. More recently, bosonic lasers have also been developed based on Bose-Einstein condensates of exciton-polaritons in semiconductor microcavities. These electrically neutral bosons coexist with charged electrons and holes. In the presence of magnetic fields, the charged particles are bound to their cyclotron orbits, while the neutral exciton-polaritons move freely. We demonstrate how magnetic fields affect dramatically the phase diagram of mixed Bose-Fermi systems, switching between fermionic lasing, incoherent emission and bosonic lasing regimes in planar and pillar microcavities with optical and electrical pumping. We collected and analyzed the data taken on pillar and planar microcavity structures at continuous wave and pulsed optical excitation as well as injecting electrons and holes electronically. Our results evidence the transition from a Bose gas to a Fermi liquid mediated by magnetic fields and light-matter coupling.

Exciton-polaritons are hybrid light-matter quasiparticles (Refs. 1, 2, 3, 4, 5, 6, 7, 8, 9). They demonstrate remarkable collective properties including polariton lasing^2^, Josephson oscillations^10^, vortices^11^, solitons^12^, the optical spin Hall effect^13^ and quenching of Zeeman splitting^14^. Clearly, microcavities present a unique laboratory rich in complex many-body processes, where fermionic and bosonic quasiparticles (electrons, holes, excitons and exciton-polaritons) coexist and interact giving rise to new phases or pseudo-phases^15^. A magnetic field is an efficient tool for switching between some of these phases, making use of the magnetic field sensitivity of both the internal structure and motion of excitons^16^. Here we present the first detailed study of the magnetic field effect on both fermionic and bosonic lasing in microcavities. So far, polariton lasers are the only existing example of bosonic lasers^17^, where coherent light is emitted spontaneously by a bosonic condensate^18^. A magnetic field was recently shown to be instrumental for the achievement of polariton lasing in electrically pumped microcavities^3^,^4^ and it also affects the second phase transition towards a photonic laser, which takes place when stimulated emission of light starts from electron-hole plasma. The switch from polariton to photon lasing^19^ is associated with the exciton Mott transition^20^: the phase transition between a bosonic gas (exciton-polariton gas) and a fermionic plasma (electron-hole plasma). Previous experimental studies evidenced a Mott transition in semiconductor quantum wells manifested by sharp changes of the photoluminescence (PL) energy and linewidth^21^,^22^.

To understand magnetic field induced transitions, we begin by examining magneto-photoluminescence spectra, shown in Fig. 1(a–e) for the case of a round pillar microcavity with GaAs/AlGaAs quantum wells for different pumping power. The external magnetic field is applied along the structure axis (Faraday geometry). The pillar microcavity structure (see Materials and Methods for details) was excited non-resonantly by short-duration light pulses focused to 1.5 μm spot diameter. These pulses create electron-hole pairs, which cool down to form excitons. These relax further and eventually form a condensate of exciton-polaritons, in the polariton lasing regime. At low pumping intensities (Fig. 1a) and relatively high magnetic fields (over 7 T) we observe a narrow and intense polariton lasing mode showing a characteristic diamagnetic shift^23^,^24^. Increasing the pumping strength or lowering the field results in dramatic modifications of the spectra (Fig. 1b): the exciton-polariton mode abruptly disappears, while a strong laser line at a higher energy emerges. This line is pinned to the cavity photon mode and is not affected by magnetic fields (see Fig. 1e). The sharp polariton to photon lasing transition is a manifestation of the abrupt excitonic Mott transition. Interestingly enough, the photon laser emission line disappears again at very weak magnetic fields at intermediate pumping power (Fig. 1c,d). The interplay between polariton and photon lasing seen in Fig. 1(a–e) shows an important and non-trivial role of the magnetic field: it is remarkable, in particular, that at the intermediate pumping power the photon lasing is suppressed both at very low and at high magnetic fields. We also note that the broadening of the polariton laser mode is due to time averaging of the photoluminescence signal in the experiment under pulsed excitation. The characteristic line narrowing at the onset of polariton lasing in the presence of magnetic field has been observed at cw pumping (see Supplementary material for details). Also ref. 24 shows characteristic narrow polariton lasing lines in the photoluminescence spectra of the same sample under cw non-resonant excitation at 0 T.

Figure 2a shows the power dependence of the spectra at *B* = 6 T. Both polariton and photon lasing regimes are clearly distinguishable and their thresholds are identified from the peak intensity dependencies on the pumping power for the lower polariton (LP) and cavity (C) photon modes (Fig. 2b). Analyzing a range of magnetic field strengths allows us to construct the phase diagram shown in Fig. 2c, which reveals a decrease of the polariton lasing threshold with magnetic field. This tendency occurs generally, across a range of pillar samples with different diameters (see Supplementary materials for details). In contrast, the behavior of the photon lasing threshold is strongly non-monotonic: initially it sharply decreases, and then increases (Fig. 2c). The switching between exciton-polariton gas and electron-hole plasma has all the features of a first order phase transition (in the context of a non-equilibrium system).

We interpret the experimental observations by the interplay of the magnetic field induced exciton Bohr radius shrinkage and field-controlled diffusion of electrons, holes and excitons. The diamagnetic effect and the phase space filling both bring the exciton transition into resonance with the cavity mode, which allows for photon lasing and fully suppresses exciton-polaritons in the course of the avalanche Mott transition. A model based on the relaxation of electrons and holes into an exciton reservoir and finally into the polariton condensate (see the scheme in Fig. 3b) at a critical exciton density is detailed in the Supplementary information and gives the dashed curves shown in the phase diagram (Fig. 2c). The coherent polariton state within our model is formed during the three-stage process illustrated schematically in Fig. 3b. The first stage is the non-resonant pumping of electrons and holes with large values of wave vectors; the second stage is the energy relaxation of charged carriers followed by the formation of an exciton reservoir, and the final stage is the formation of polaritons followed by their resonant scattering to the ground state of the condensate. The first two processes can be described by the following set of dynamical diffusion equations:


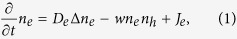



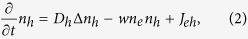



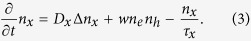


Here *n*_*e*_, *n*_*h*_ and *n*_*x*_ are the densities of electrons, holes and excitons, respectively; *D*_*i*_ are the diffusion coefficients, *w* describes the exciton formation rate; *J*_*e*_ and *J*_*h*_ are the pump rates for electrons and holes and τ_x_ is the exciton lifetime. The formation of excitons in our model is described by the term *wn*_*e*_*n*_*h*_. Since in a strong coupling regime non-radiative losses are negligible, we assume that excitons decay mainly due to radiative processes, so that *τ*_*x*_ corresponds to the radiative lifetime.

To describe the formation of a condensate the equation (3) should be supplemented with the scattering terms:


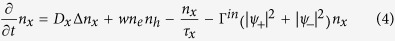


Here Γ^*in*^ is the transition rate from the reservoir to the condensate. The equations for the condensate wavefunctions ψ_±_ read:


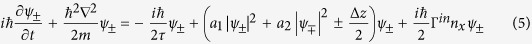


Here *m* is the effective mass of a polariton at zero wave vector, *τ* is a lifetime of the condensate, Δ*z* is the Zeeman splitting and *α*_1,2_ are polariton-polariton interaction constants for the parallel and anti-parallel spin configurations. We note that in the present equations we neglect the drift terms, arising from the different spatial distribution of electrons and holes due to the possible difference in the diffusion coefficients *D*_*e,h*_. Comparison with more accurate simulations (including the drift currents) showed the same behaviors for the phase diagrams with only minor quantitative differences, therefore we will further use *D*_*e*_ = *D*_*h*_ ≡ *D* and omit the possibility for electrical currents inside the structure.

The decrease of the polariton laser threshold with magnetic field is due to the suppression of in-plane diffusion of electrons and holes away from the excitation spot, because the magnetic field causes an orbital motion. The larger carrier concentration caused by suppressed diffusion also impacts the excitonic Mott transition, which occurs around^25^


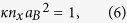


where *n*_*x*_ and *a*_*B*_ are the exciton concentration and Bohr radius, respectively, and *κ* is a coefficient depending on the structure geometry. The increase of *n*_*x*_ with low magnetic fields leads to the decrease of the photon lasing threshold seen in Fig. 2c. At high fields, the shrinkage of the exciton Bohr radius^26^ reduces the exciton lifetime, leading to a lower exciton concentration and increased critical pumping power.

In order to check the universality of the observed effects, we also studied magneto-photoluminescence spectra of planar microcavity samples (see Supplementary materials for more details), which are shown in Fig. 3 for a non-resonant CW excitation. The threshold to polariton lasing was again strongly sensitive to the magnetic field (Fig. 3a) and also to the size of the excitation spot, as the phase diagrams in the lower panels of Fig. 3 show. For a small (10 μm) spot, the threshold non-monotonically depends on the field (Fig. 3d). For larger spots a steady and monotonic increase with magnetic field is recovered (Fig. 3e,f). This behavior is consistent with our kinetic model which predicts a qualitatively similar dependence of the threshold of the magnetic field. At zero fields, the exciton concentration is strongly diluted by carrying diffusion if the pump spot is narrow (see Figs 3c and S2). On the other hand, for large spots, the diffusion has little effect on the exciton concentration, which is why the suppression of diffusion by a magnetic field does not affect the threshold of polariton lasing so strongly. The threshold increase with a magnetic field observed for all spot sizes is again due to the shrinkage of the exciton Bohr radius. The predictions of our simple theoretical model deviate from the data, especially for the small spot sizes. This may be caused by a multitude of many-body effects in the complex Bose-Fermi system we study. One can mention, in particular, the repulsion of exciton-polaritons from the exciton reservoir and the electron-hole plasma.

Polariton lasers with electrical injection strongly differ dynamically from optically pumped lasers. Indeed, electron and hole injection is nearly homogeneous over the whole area of our sample, which is why diffusion effects play little role. On the other hand, the life-time of excitons in the structure is shortened in the presence of the magnetic field^27^ which leads to the increase of the polariton lasing threshold (Fig. 4a).

Figure 4b shows the polariton and photon lasing thresholds at *B* = 5 T as functions of temperature. Both thresholds decrease in agreement with the theory, which accounts for acceleration of the acoustic phonon relaxation processes leading to electron and hole relaxation to the exciton reservoir and exciton relaxation to the polariton condensate. Figure 4c shows both polariton and photon lasing thresholds seen in the linewidth dependence on the injection current. Positions of both thresholds are consistent with the theoretical prediction shown in Fig. 4b.

In conclusion, the polariton and photon lasing thresholds were shown to depend strongly on magnetic field. Particularly, the suppression of charged carrier diffusion may lead to reduction of the polariton lasing threshold. For comparison, photon lasing in the presence of a magnetic field was studied in the same samples, and is governed by the excitonic Mott transition^28^. All the observed experimental features are described within a uniform model based on coupled diffusion equations for electrons, holes and excitons and the Gross-Pitaevskii equation for exciton-polariton condensates. Our results manifest a high potentiality of low threshold polariton lasers for spin-based logic applications^28^,^29^.

## Methods

### Sample design and fabrication

**Figure 1:**
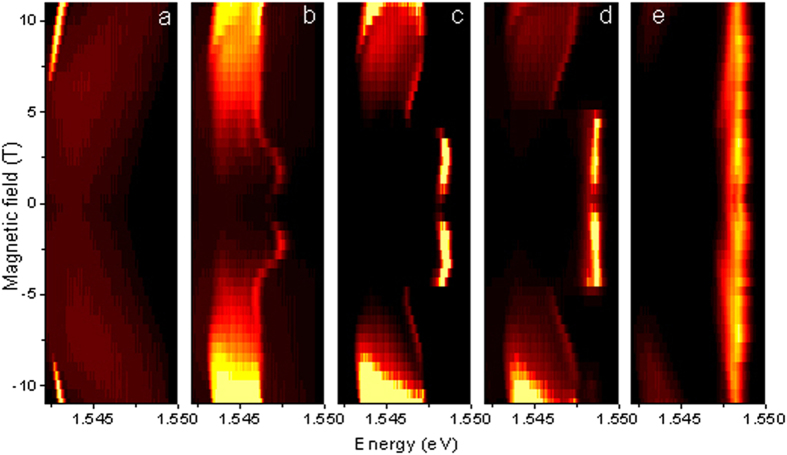
Magneto-photoluminescence of the 5 *μ*m diameter micropillar sample. Panels represent different pumping powers: 0.09 mW (**a**), 0.58 mW (**b**), 0.85 mW (**c**), 1 mW (**d**) and 1.5 mW (**e**). The excitation laser spot was of 1.5 μm size. Both polariton lasing (**a**) and a very sharp transition to photon lasing (**c**–**e**) are observed.

**Figure 2:**
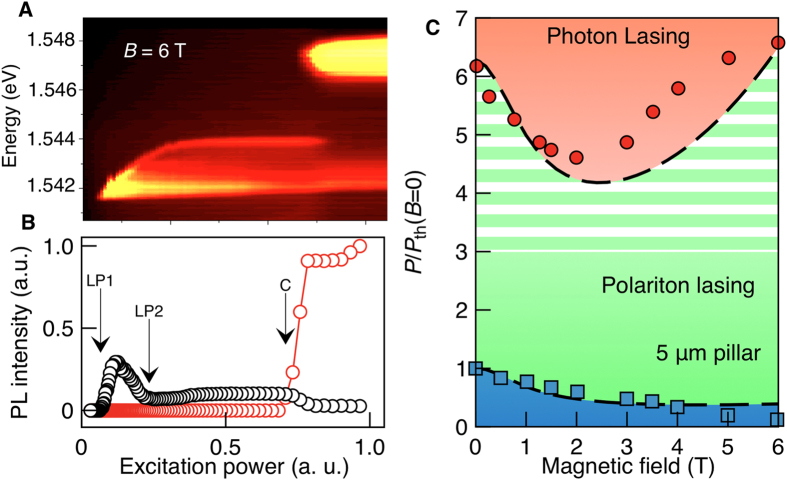
Phase transitions in micropillar samples. (**a**,**b**) Emission pattern and the integrated PL intensity of the 5 μm round pillar at *B* = 6 *T*. In (**b**) spectral integration has been performed over the polariton lasing peak (black circles) and photon laser peak (red circles), for details see Supplementary material. The arrows indicate the onset and offset of the polariton laser (LP1 and LP2) and the photon laser threshold (C). (**c**) Phase diagram of a 5 μm round pillar. The red circles correspond to the photon lasing threshold (C), the blue squares show the onset of polariton lasing (LP1). The offset of polariton lasing (L2) is shown only indicatively by the boundary between green color and white-green stripes. We cannot extract this threshold from the data with a high accuracy. Note that the polariton lasing transition for zero field reported in Fig. 2c corresponds to a pump power intermediate between those considered in Fig. 1a,b. The lines show the results of simulation. White horizontal bands mark the polariton gas regime, beyond the offset of polariton lasing.

**Figure 3:**
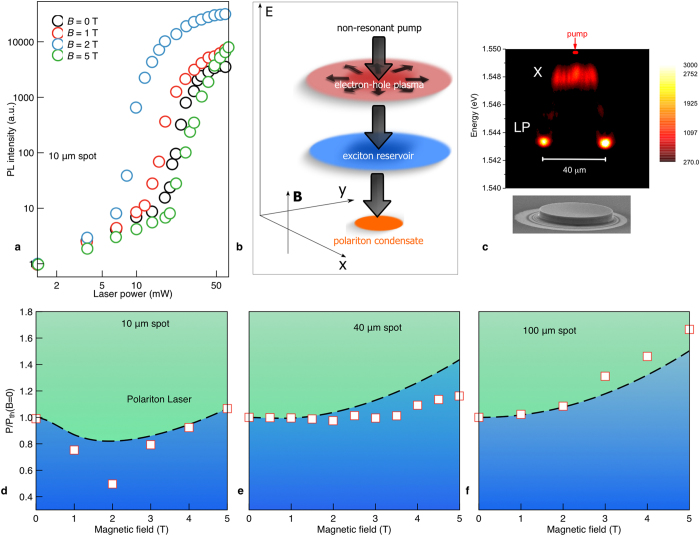
Magneto-polariton lasing in a planar microcavity sample. (**a**) Power dependence of the integrated PL intensity at different magnetic fields for a 10 μm spot. (**b**) Scheme of non-resonant polariton formation. (**c**) Spatially resolved PL spectra in a large pillar showing the diffusion of excitons over 10 μm away from a 2 μm spot. (**d**–**f**) Phase diagrams of polariton emission for excitation spot sizes: *d* = 10, 40 and 100 μm. The white squares and dashed curves show the experimental and theoretical data, respectively.

All structures have been grown by molecular beam epitaxy. The planar sample is a high-finesse Q > 16000 microcavity formed by 5/2 λ Al_0.3_Ga_0.7_As cavity surrounded by 32 (35) period AlAs/Al_0.15_Ga_0.85_As top (bottom) DBR mirrors. Four sets of three 10 nm GaAs quantum wells are embedded inside the cavity at the antinodes of the electric field producing a Rabi splitting of Ω_R_ = 9.2 meV. For the micropillars studies, reactive ion etching has been applied to sculpt circular mesas with diameter ranging from 1 to 40 μm. The data shown in Figs 1 and 2 are taken at the positive exciton-photon detuning of about 2 meV. The data in Fig. 3 are taken at the negative detuning of 7 meV. The sample for a polariton laser with electrical injection is the same as in ref. 16.

### Experimental set-up

The optically excited spectra were pumped by a Ti:Sa laser with pulse duration of 2 picoseconds and energy 1.62 eV, which corresponds to a range of transparency in the Bragg mirrors. Time integrated micro PL spectra of the pillar samples were recorded by a CCD detector in magnetic fields up to 11 T in the Faraday geometry with spatial resolution of about 1 μm. The planar microcavities were mounted in a gas flow sample chamber kept at T = 3 K of superconducting cryostat operating in magnetic fields up to 5 T. The PL signal was integrated in a solid angle determined by numerical aperture (NA) of the collimating lens (NA = 0.5 for 10 μm spot and NA = 0.08 for 100 μm spot). All PL experiments were performed in back scattering geometry and at about normal incidence of the light on the sample surface.

## Additional Information

**How to cite this article**: Kochereshko, V. P. *et al*. Lasing in Bose-Fermi mixtures. *Sci. Rep*. **6**, 20091; doi: 10.1038/srep20091 (2016).

## Supplementary Material

Supplementary Information

## Figures and Tables

**Figure 4:**
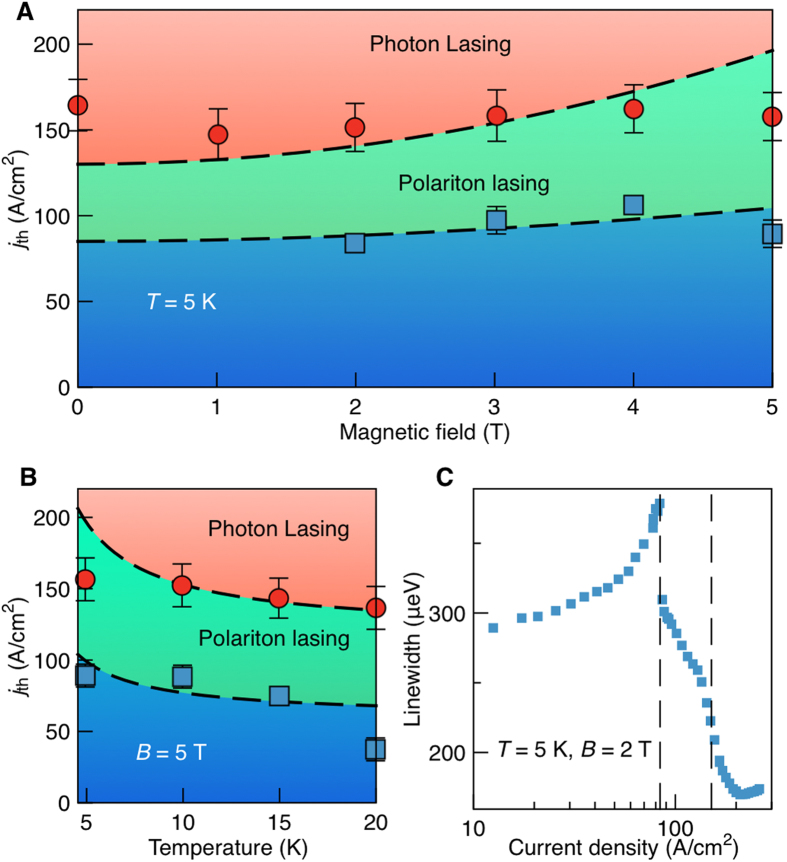
Phase diagrams of the electrically pumped polariton laser. (**a**,**b**) Magnetic and temperature dependent phase diagrams. Dashed lines show the results of our kinetic modelling. (**c**) Linewidth of the PL emission as a function of injection current, with the onset of polariton and photon lasing marked by dashed lines. The laser structure represented a pillar of 20 μm diameter, see for details ref. 3.
