# Supplementary material for: Lasing in Bose-Fermi mixtures
Source: Sci Rep. 2016 Jan 29;6:20091. doi: 10.1038/srep20091 (PMC4731768; doi:10.1038/srep20091)
Supplement: Supplementary Information [file srep20091-s1.pdf]

Lasing in Bose-Fermi mixtures

Vladimir P. Kochereshko[1,2], Mikhail V. Durnev[1,2], Lucien Besombes[3], Henri Mariette[3], Victor F. Sapega[1,2], Alexis Askitopoulos[4], Ivan G. Savenko[5,6], Timothy C.H. Liew[7], Ivan A. Shelykh[7], Alexey V. Platonov[1,2], Simeon I. Tsintzos[9], Z. Hatzopoulos[9], Pavlos G. Savvidis[8,9], Vladimir K. Kalevich[1,2], Mikhail M. Afanasiev[1,2], Vladimir A. Lukoshkin[1,2], Christian Schneider[10], Matthias Amthor[10], Christian Metzger[10] , Martin Kamp[10], Sven Hoefling[10], Pavlos Lagoudakis[4], Alexey Kavokin*[1,4]

[1] Spin Optics Laboratory, Saint-Petersburg State University, 1, Ulianovskaya, 198504, St-Petersburg, Russia.

[2] Ioffe Physical-Technical Institute, Russian Academy of Sciences, 26, Politechnicheskaya, 194021, St-Petersburg, Russia.

[3] Institut Néel, CNRS/UJF 25, avenue des Martyrs - BP 166, Fr-38042 Grenoble Cedex 9, France.

[4] Faculty of Physical Sciences and Engineering, University of Southampton, Highfield, Southampton, SO171BJ, UK.

[5] Science Institute, University of Iceland, Dunhagi-3, IS-107, Reykjavik, Iceland.

[6] Department of Applied Physics/COMP, Aalto University, PO Box 14100, 00076 Aalto, Finland.

[7] Division of Physics and Applied Physics, Nanyang Technological University 637371, Singapore.

[8] Department of Materials Science & Technology, University of Crete, Greece.

[9] IESL-FORTH, P.O. Box 1527, 71110 Heraklion, Crete, Greece.

[10] Technische Physik and Wilhelm-Conrad-Röntgen-Research Center for Complex Material Systems, Universität Würzburg, D-97074 Würzburg, Am Hubland, Germany.

*Corresponding author: pavlos.lagoudakis@soton.ac.uk

SUPPLEMENTARY MATERIAL

Theoretical model

Low and high pumping regimes

Let us, first, focus on the set of equations (1) for the exciton reservoir assuming the onset of polariton lasing at $n_x = n_x^{(th)}$, where $n_x^{(th)}$ is a critical exciton concentration corresponding to the population of polariton ground state equal to unity. In the following we assume that the electron and hole components are pumped with equal rates by the Gaussian-shaped laser beam, $J_e = J_h \equiv Je^{-r^2/R^2}$. The absence of the drift currents means equal concentrations for electrons and holes, $n_e = n_h \equiv n$, satisfying the following equation in the stationary limit:

$$D\Delta_n - wn^2 + J(r) = 0 \qquad (S1)$$

Nonlinear Eq. (4) can be solved numerically, however a simple variational procedure with the carrier density taken in the form $n(r) = n_0 e^{-r^2/a^2}$ gives a good approximation preserving all the physical insight. Moreover, there are two limiting cases when Eq. (S1) allows for analytical solutions. The first one corresponds to a low pumping regime: in that case one deals with low concentrations and the nonlinear term in Eq. (S1) is small. The effective lifetime of a particle is then $\tau = 1/(wn_0)$, and the spread of carrier density is $a = \sqrt{D\tau}$. Integration of Eq. (S1) over space domain gives: $a = D/\left(R\sqrt{wJ}\right)$. This effective radius obviously should be greater than the radius of the pump spot, R, so that $\alpha \gg R$ and $J \ll J^* = 2D^2/(wR^4)$. The excitonic density at $r = 0$ is $n_x(0) = wn_0^2\tau_x$ (if one totally neglects the diffusion of excitons, i.e. sets $D_x \equiv 0$) and it behaves quadratically with the pump intensity:

$$n_x^{lowpump}(0) \propto \frac{wR^4}{2D^2} J^2 \tau_x = \frac{J^2}{J^*}\tau_x \, . \qquad (S2)$$

In the opposite case of a high pump regime, we can neglect the diffusion term in Eq. (S1) resulting in a very short, linear in pump, expression for the exciton density:

$$n_x^{highpump}(0) = J\tau_x \, . \qquad (S3)$$

Since the transition between the discussed regimes is defined by J^* which is inversely proportional to the fourth power of R, one should expect that the excitonic density distribution dependence on the pump power with and without a magnetic field significantly depends on the excitation spot radius. This effect is illustrated in Fig. S1a, where $n_x(0)$ is plotted for two spot

radii different by one order of magnitude. One can see that for the sharper spot (red curve) $J*$ significantly increases. Thus, for the fixed exciton density we switch between the high-pumping regime (black curve) and the low-pumping regime (red curve).

Application of a magnetic field alters parameters D and τ_x. Magnetic field leads to suppression of the diffusion of charged carriers which can be described using the Einstein relation and the Hall expression for conductivity:

$$D(B) = \frac{k_B T \mu_0 / e}{1 + B^2/B_0^2}\,, \quad B_0 = \frac{c}{\mu_0}\ . \qquad (S4)$$

Here $\mu_0 = e\tau_p/m$ is the mobility of electron/hole gas at B = 0 and T is its temperature. The experimental data show that T might be significantly higher than the lattice temperature (e.g. T ≈ 50 K). Excitonic lifetime decreases with the magnetic field due to the decrease of the exciton Bohr radius ($\tau_x \propto a_B^2$ in 2D structures [6], see Fig. S1b). This dependence has been analyzed variationally in Ref. 26 to the main paper. The radiative life-time is inversely proportional to the exciton oscillator strength. Its dependence on the magnetic field can be approximated by a Lorentzian:

$$\tau_x(B) = \frac{\tau_0}{1 + B^2/B_1^2} \qquad (S5)$$

with B_1 sufficiently larger than B_0 [7].

It becomes obvious now, that for the two regimes discussed above one should expect completely different behavior of excitons' density in the center of the spot as a function of magnetic field. Indeed, in the high pumping regime, $n_x(0) \propto \tau_x(B)$ decreases monotonously as a function of magnetic field, while in the low pumping regime, $n_x(0) \propto \tau_x(B)/D^2(B)$ which (in the case $B_1 > B_0$) results in the enhancement of exciton formation at r = 0. Further, since parameter $J*$ depends on the magnetic field via the diffusion coefficient, the magnetic field acts as a switch between the two regimes leading to a non-monotonous behavior of the excitons' density. This is illustrated in Fig. 3c in the main text of the article.

Results of calculations and parameter sets used

As it has been already mentioned, the onset of polariton lasing is described by a condition $n_x = n_x^{(th)}$, where $n_x^{(th)}$ can be set manually. The photon lasing onset coincides with the Mott transition in the system and it is described by the following condition [8]:

$$\kappa n_x a_B^2 = 1\,, \qquad (S6)$$

where κ is the coefficient of the order of unity. In the simulations we set $\kappa = 1$, while its exact value is not essential for the magnetic field dependence of the Mott transition threshold. Note that the absolute values of electron and hole concentration cannot be experimentally measured with a good accuracy. Using those two conditions, one can find the threshold pump intensity as a function of magnetic field. This function is plotted in Fig. 2c, Fig. 2d and Fig. 3d-f in the main text.

The experimental values for w found in literature are: $w = 6 \pm 2\, cm^2/s$ [9], $w = 15\, cm^2/s$ [1], $w \geq 0.5\, cm^2/s$ [10]. The theoretical calculations (accounting for relaxation on optical and acoustic phonons [2]) result in $w = 0.01\, cm^2/s$.

The following sets of parameters were employed to calculate the phase diagrams shown in the main text:

1. **Fig. 2c**

$D_0 = 1000\, cm^2/s\,, B_0 = 1\text{T}, B_1 = 9\text{T}, w = 1\, cm^2/s\,, n_{th} = 5 \times 10^{10} cm^{-2}, a_B(B = 0) = 13nm$

2. **Fig. 2d**

$D_0 = 1500\, cm^2/s\,, B_0 = 2.5\text{T}, B_1 = 10\text{T}, w = 0.1\, cm^2/s\,, n_{th} = 5 \times 10^{10} cm^{-2}$

3. **Fig. 3d-f**

$D_0 = 900\, cm^2/s\,, B_0 = 1\text{T}, B_1 = 7\text{T}, w = 1\, cm^2/s\,, n_{th} = 3 \times 10^{9} cm^{-2}$

To account for the coupling between the excitonic reservoir and the exciton-polariton condensate

To investigate the role of the magnetic field on the formation of the exciton-polariton condensate from the reservoir of excitons, we used numerical analysis of the coupled Eqs. (1), (2), (3). The results of modelling of the condensate real-space profile, $|\psi|^2$, are presented in Fig. S2. One can see that, in agreement with the calculations for the excitonic reservoir, a magnetic field leads to localization of the condense density. The calculated phase diagrams describing polariton lasing show the following behavior: monotonous threshold increase in the case of the wide laser spot and non-monotonous behavior in the case of the sharp focusing. Thus, it is important to mention, that this accurate model validates the simple calculations presented above which account for the evolution of the excitonic component only.

Equations for the laser with current injection

The situation is quite different if we consider an electrically pumped laser. In this case an electron and a hole are excited in different spots of the sample, thus leading to small values of the parameter w. We will assume that in our sample the electrical injection is homogeneous along the quantum well planes meaning that the diffusion is neglected in the case of current injection. On the other hand, there are lots of possible mechanisms of electron and hole decays not leading to the exciton formation (including the ‘fly-through’ of the particles, non-radiative processes at the pillar surface, etc.), so that the non-radiative decay times τ_e and τ_h should be introduced. The kinetic equations similar to Eq. (1) therefore read:

$$\frac{\partial}{\partial t} n_e = -\mathrm{w} n_e n_h - \frac{n_e}{\tau_e} + J_e$$

$$\frac{\partial}{\partial t} n_h = -\mathrm{w} n_e n_h - \frac{n_e}{\tau_e} + J_{eh}$$

$$\frac{\partial}{\partial t} n_x = \mathrm{w} n_e n_h - \frac{n_x}{\tau_x} \qquad (S7)$$

In the stationary limit, we obtain the algebraic system of equations, the solution of which reads:

$$n_e = -\frac{1}{2w\tau_h} - \frac{1}{2}(J_h - J_e)\tau_e + \sqrt{\frac{1}{4}\left[\frac{1}{w\tau_h} + (J_h - J_e)\tau_e\right]^2 + J_e \frac{\tau_e}{w\tau_h}}\,, \qquad (S8a)$$

$$n_h = n_e \frac{\tau_h}{\tau_2} + (J_h - J_e)\tau_h \quad . \qquad (S8b)$$

We will further make some simplifications, namely: $\tau_e = \tau_h \equiv \tau$ and$J_e = J_h \equiv J$, so that $n_e = n_h \equiv n$. In a realistic limit of small carrier lifetimes, $Jw\tau^2 < 1$, the carrier concentration is $n = J\tau$, and for the exciton density one obtains:

$$n_x = wJ^2\tau^2\tau_x.$$

We will assume a usual dependence of τ_x on magnetic field [see Eq. (S5)], which gives the following expressions for the polariton and photon thresholds:

$$J_{th}^{(pol)} B = J_{th,0}\sqrt{1 + \frac{B^2}{B_1^2}}\,, \qquad (S9a)$$

$$J_{th}^{(pol)} B = \frac{J_{th,0}}{\sqrt{n_{th} a_B^2(0)}}\left(1 + \frac{B^2}{B_1^2}\right), \quad (S9b)$$

Here $J_{th,0} = \sqrt{n_{th}/(w\tau_x(0)\tau^2)}$ is the polariton threshold at zero magnetic field. The calculated

phase diagram is presented in Fig. 4a in the main text with $J_{th,0} = 85\ A/cm^2$, $B_1 = 7$ T and $n_{th,0}a_B^2(0) = 0.65$.

The temperature dependence of lasing thresholds is ruled by the strongly temperature-dependent exciton formation rate, w. We assume that the exciton formation rate in the low-temperature region increases with temperature, following: $w(T) = w_0\exp(-T_0/T)$, where T0 is some effective temperature. It leads to a decrease of the thresholds which is illustrated in Fig. 4b of the main text. The value of T_0 is chosen T_0 = 5 K.

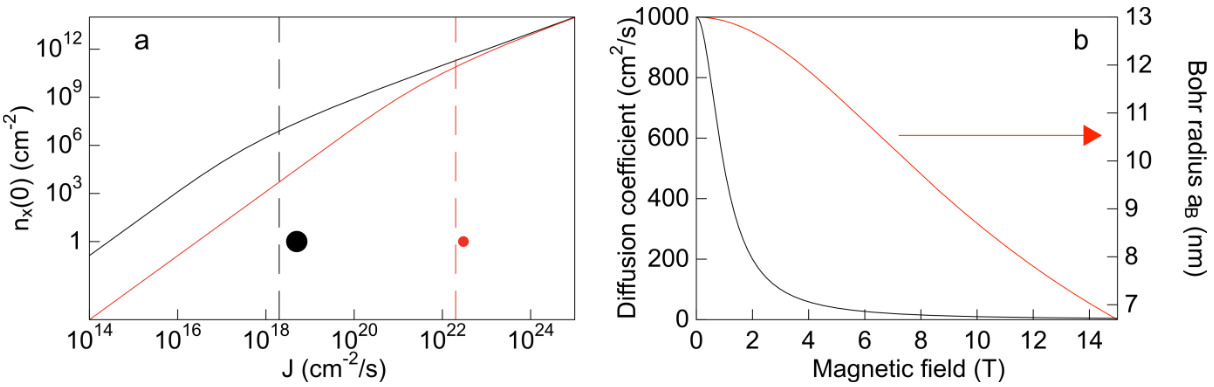

Figure S 1: **Switch between the linear and nonlinear regimes.** (a) Excitonic density in the center of the spot as a function of pump intensity. Red curve corresponds to a sharper focusing, the radii are different by one order of magnitude. The dashed lines indicate the position of *J**. (b) Bohr radius and diffusion coefficient dependences on magnetic field as used in calculations presented in Fig. 2c in the main text.

Supplementary experimental data

<

This section summarizes additional experimental data not presented in the main text of the Letter. Let us start with the micropillar samples. Figure S2 shows that PL peak intensity, energy and full width at half maximum (FWHM) in a pillar microcavity sample at the magnetic field of 5T. The data were collected from a AlAs/GaAs pillar microcavity with a diameter of 20 µm and 4 embedded InGaAs QWs (Ref. 12) .PL spectra were recorded under non-resonant cw excitation, and the device was excited at the first Bragg minimum above the stopband. One can clearly see the threshold for polariton lasing manifested in the factor of 1000 increase of the PL intensity,

line narrowing and the characteristic blue shift. Figure S3 shows micropillar PL spectra of under pulsed excitation at the on-set and off-set of polariton lasing and at the maximum of polariton laser intensity. Figure S4 shows the PL spectra just below and just above the threshold to photon lasing. These spectra have been used to obtain the points in Figure 2(b) of the main text. We note that while the spectral range chosen for integration does have influence on the resulting value of the integrated intensity, of course, the positions of both polariton and photon laser thresholds extracted from the spectrally integrated data are essentially independent on the chosen spectral integration range. Figure S5 shows the spatial shape of polariton condensates in a pillar cavity in the absence and in the presence of the magnetic field. One can see that the field induces a supplementary localization of the condensate. Also, the spatial disorder effect on the shape of the condensate is quite pronounced. The disorder induced localization of polariton condensates has been studied e.g. in[11] . Figure S6 presents the data on the 8 μm diameter pillar manifesting the role of magnetic field on the in-plane motion of the carriers: due to diffusion quenching the center region of the pillar is depleted with carriers at high magnetic fields which results in lasing suppression (indicated by the arrow). On the other hand, the emission intensity increases monotonously when the pillar is excited at the center. Fig. S7 presents the experimental data on thresholds to polariton lasing measured for two micropillar samples with diameters 8 and 14 μm (the data on a 5 μm sample is presented in the main text). Both pillars clearly illustrate the suppression of diffusion by magnetic field and show the threshold behavior which correlates well with the theory. However the initial rise of the threshold in the 14 μm sample is still unclear and needs further investigation.

The additional data on the planar samples is summarized in Figs. S8 and S9. Fig. S8 shows PL spectra of the sample with a planar microcavity for varying pump intensities at a fixed magnetic field B = 5 T and negative detuning $\delta = -7$ meV. One can see that the onset of a polariton lasing is clearly observed for both excitation spots. We conjecture that pronounced PL peaks observed at the high energy side of the lower polaritonic branch for a 10 μm spot and above threshold power originate from the cavity modes characterized by large k-vectors. Indeed, the lens with a larger NA (10 μm spot) integrates the PL signal in a wider k-space than that with a smaller NA (100 μm spot). Our additional study demonstrates that the phase diagrams (see Fig. 3d-f in the main text) are also sensitive to the detuning. Figure S9 shows the phase diagrams obtained for large 100 μm (upper panel) and small 10 μm (lower panel) spots measured at optimal negative detuning δ = −7 meV (the highest efficiency of lasing) and δ = −3.3 meV. One can see

that deviation of the detuning from the optimal to less negative values significantly decreases the effect of the magnetic field on threshold for the large spot. In contrast the effect of detuning variation for the small spot is almost negligible.

Figure S10 presents the Input-output characteristics and the emission linewidth of the electrically pumped microcavity sample. The polariton laser threshold is manifested by sharp increase in the intensity at 2 T and 4 T as shown in Fig. S8a, while the weak coupling laser threshold is characterized by a smooth S-curve at higher current values. The onset of polariton lasing is also accompanied by a sharp drop in the linewidth (see Fig. S8b).

Finally, figure S11 shows the data on the photoluminescence of the 8- μm diameter micropillar taken at the same sample as the data in Figures 1 and 2 of the main text. The threshold dependence on the magnetic field is shown in the lower panel to this figure. One can clearly see that lasing persists at cw pumping. The threshold dependence on the magnetic field is essentially the same at cw pumping (Figure S11) and pulsed excitation (Figure S7).

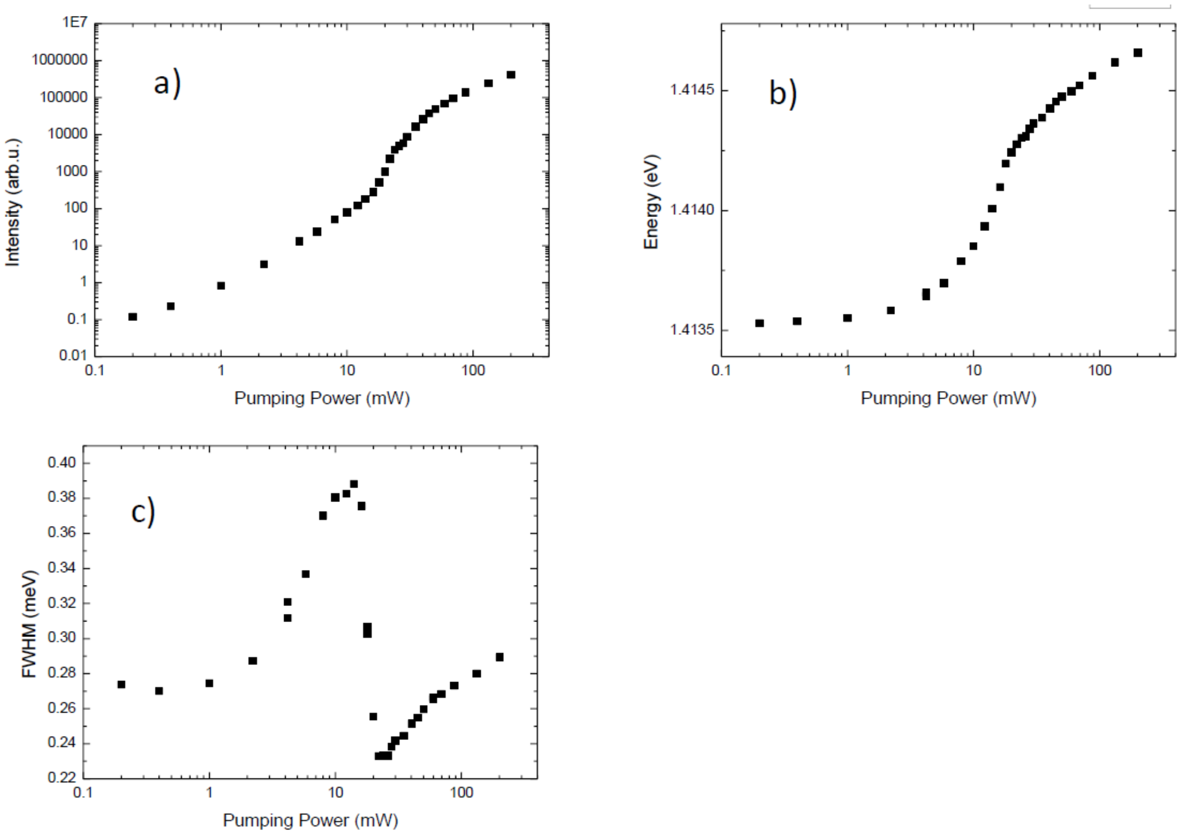

Figure S2. The PL intensity (a), peak energy (b) and full width at half maximum (c) measured at a pillar microcavity of 20 μm diameter measured at the non-resonant cw excitation at the magnetic field of 5T.

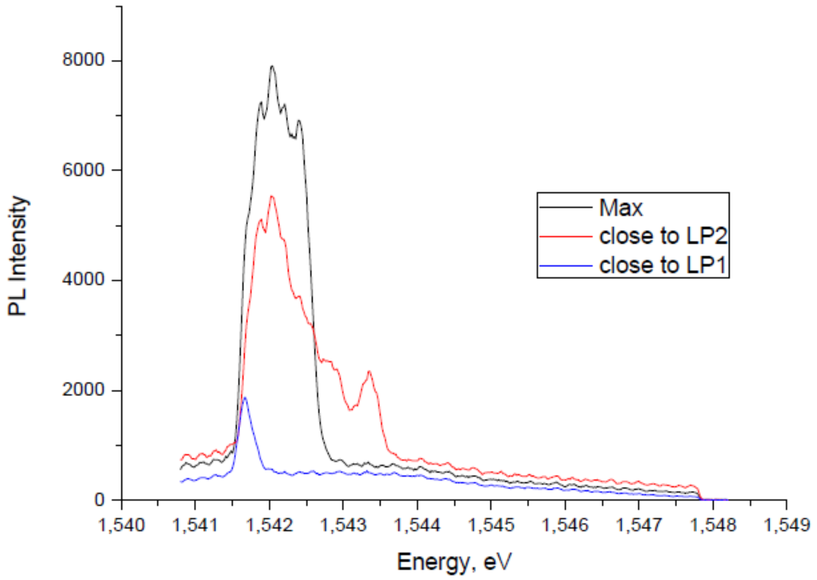

Figure S3 Characteristic PL spectra of a micropillar polariton laser taken at different pumping intensities at the onset of polariton lasing (blue curve), offset of polariton lasing (red curve) and at the maximum intensity of polariton lasing (black curve). The spectral integration in Figure 2(b) of the main text has been done within the area corresponding to the peak at the black curve.

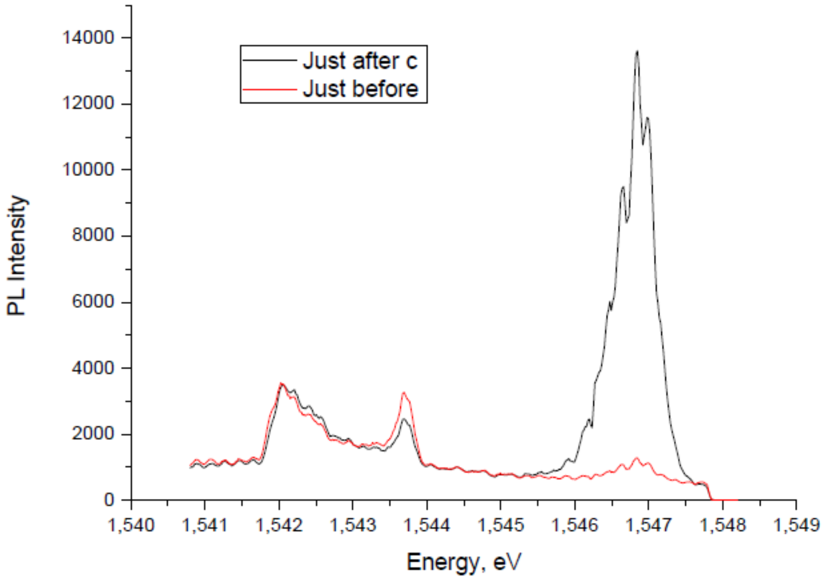

Figure S4. PL spectra of the pillar sample just before and after the threshold to photon lasing (red and black curves, respectively). The integration in Figure 2(b) of the main text has been performed in the spectral area of the black peak.

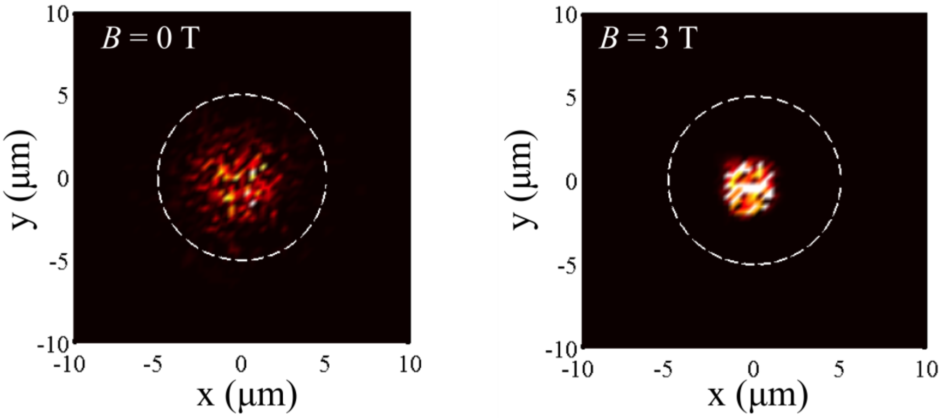

Figure S 5: **Real-space profiles of the condensate density $|\psi(x,y)|^2$.** One can see, that application of a magnetic field leads to a stronger localization of the condensate. The excitation spot diameter is 1.5 μm. The other parameters used are: $\boldsymbol{\Gamma_{in} \approx 0.005\ \mu\mathrm{eV},\ w = 1\,cm^2/s,\ a_B = 10\ \mathrm{nm}, \tau_x = 100\ \mathrm{ps}, \tau = 10/(1 + B^2/B_1^2)\ \mathrm{ps},\ B_0 = 1\ \mathrm{T},\ B_1 = 7\ \mathrm{T},\ D_0 = 1000\ \mathrm{cm^2/s},\ D_x = 100\ \mathrm{cm^2/s}}$

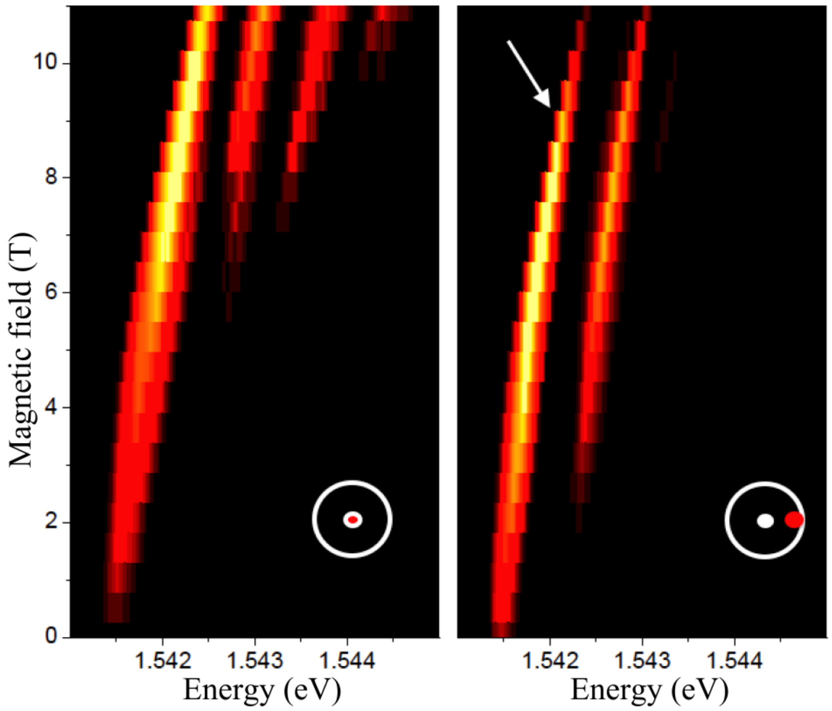

Figure S 6: **Magneto PL patterns of the 8 μm diameter micropillar sample in the polariton lasing regime.** The pillar is excited at the center (left panel) and at the edge (right panel), while the signal is detected from the center in both cases.

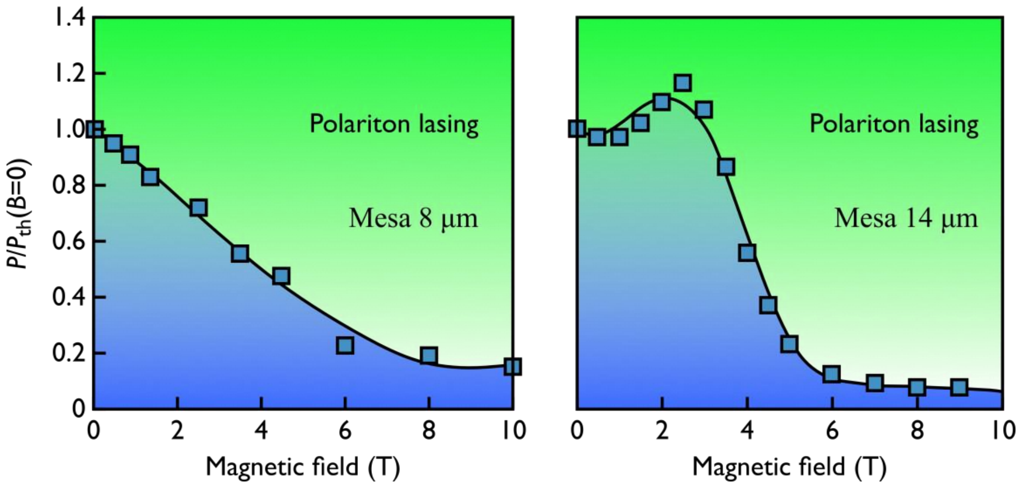

Figure S 7: **Experimentally observed phase diagrams for polariton emission of the micropillar samples.** (Left panel) pillar diameter is 8 μm, (right panel) pillar diameter is 14 μm.

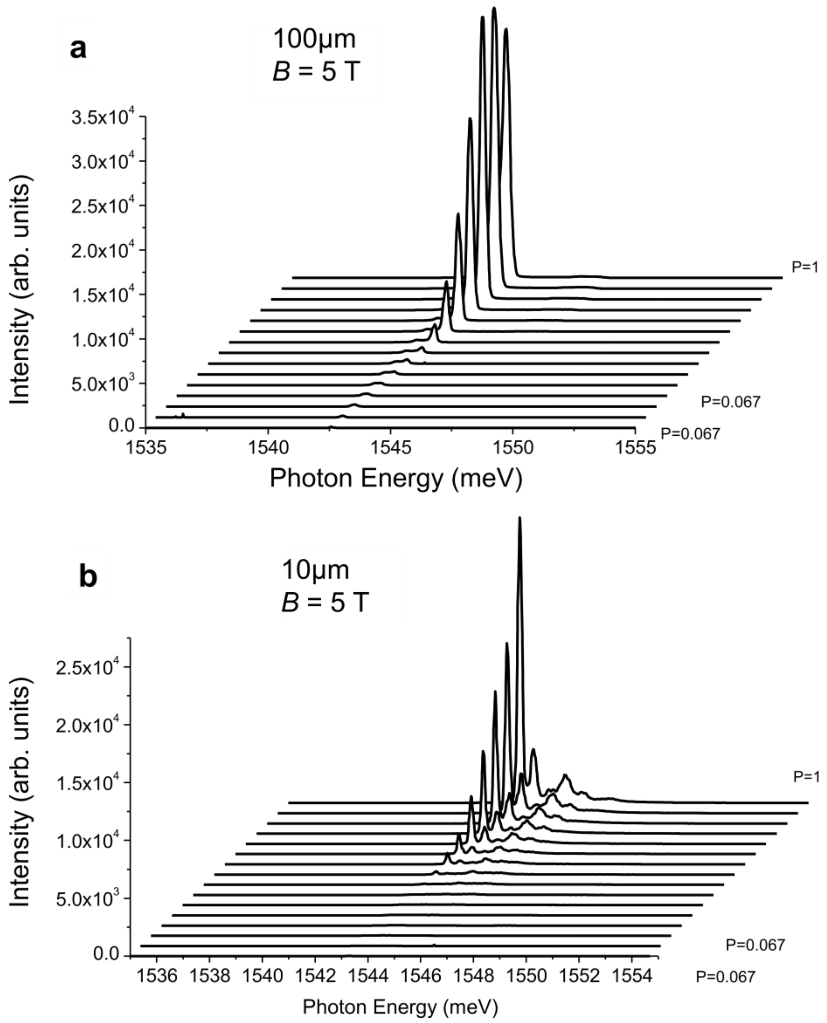

Figure S 8: **PL spectra of the planar microcavity illustrating the onset of a polariton lasing.** (a)Excitation spot diameter is 100 μm, (b) excitation spot diameter is 10 μm.

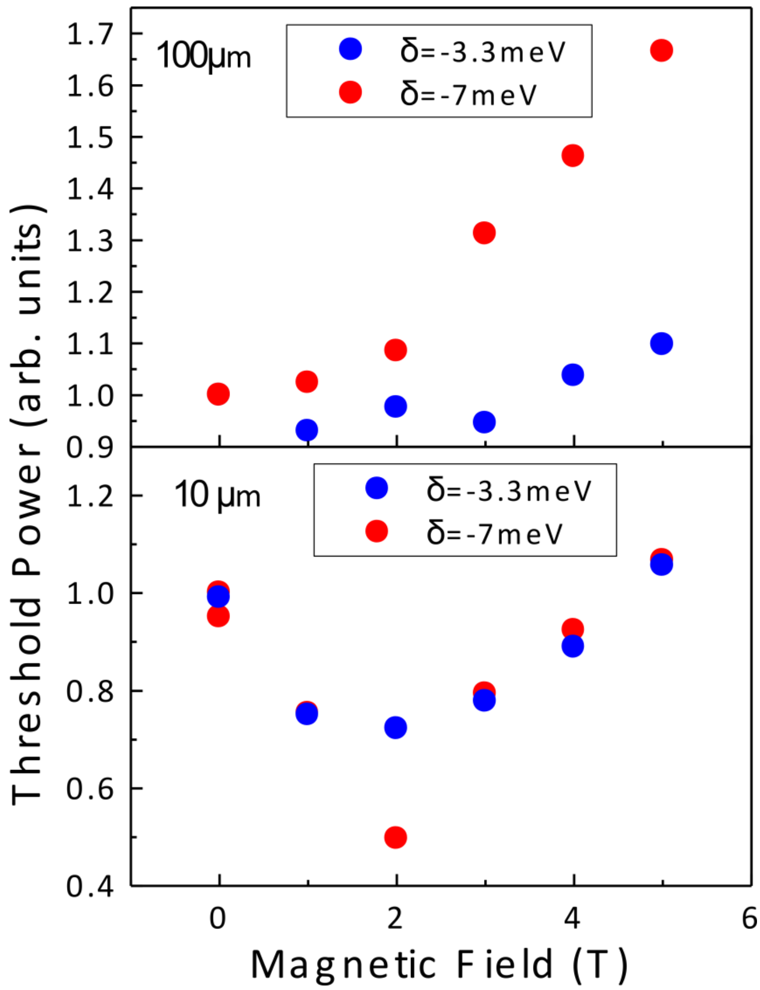

Figure S 9: **Polariton threshold of the planar microcavity sample.** The data for two different diameters of the excitation spot and two values of detuning is presented.

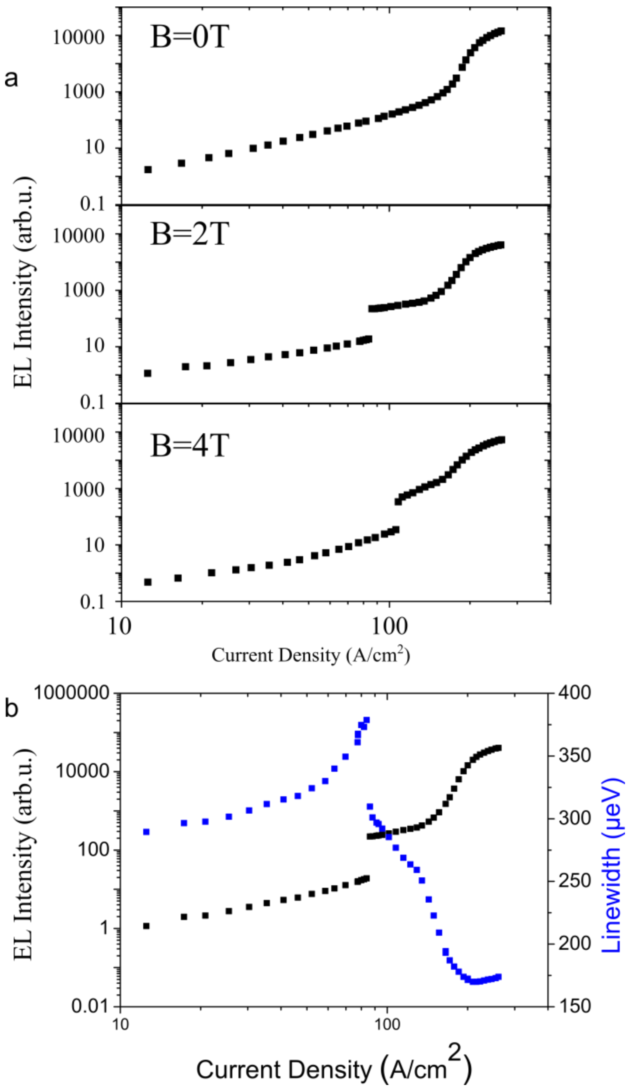

Figure S 10: **Input-output characteristics and the emission linewidth of the electrically driven polariton laser.** (a) Input-output characteristics at a magnetic field of 0T, 2T and 4T. (b) Input-output characteristics and the emission linewidth at 2T.

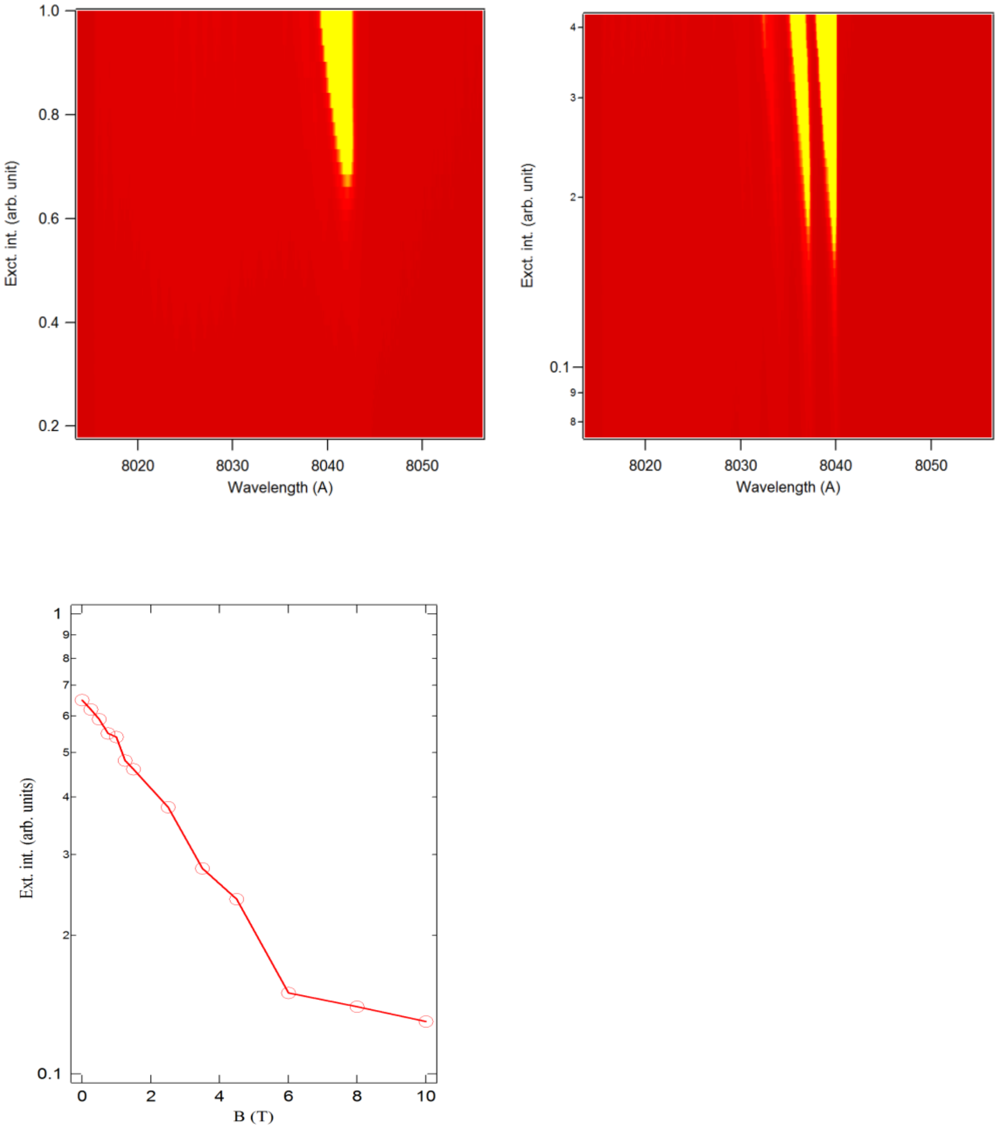

Figure S11. The photoluminescence spectra of a 8- µm diameter pillar microcavity from the same sample as one characterised in Figure 1 and 2 of the main text. The spectra have been taken at cw excitation. The upper left image is obtained at 0T, while the upper right plot corresponds to the magnetic field B=8 T. The lower panel shows the polariton laser threshold dependence as a function of magnetic field obtained from the data taken at cw pumping.

1. Robart, D. *et al.* Dynamical equilibrium between excitons and free carriers in quantum wells. *Solid State Communications* **95,** 287–293 (1995).
2. Piermarocchi, C., Tassone, F., Savona, V., Quattropani, A. & Schwendimann, P. Exciton formation rates in GaAs/AlxGa1-xAs quantum wells. *Phys. Rev. B* **55,** 1333–1336 (1997).

3. Carusotto, I. & Ciuti, C. Probing Microcavity Polariton Superfluidity through Resonant Rayleigh Scattering. *Phys. Rev. Lett.* **93,** 166401 (2004).

4. Shelykh, I. A., Rubo, Y. G., Malpuech, G., Solnyshkov, D. D. & Kavokin, A. Polarization and Propagation of Polariton Condensates. *Phys. Rev. Lett.* **97,** 066402 (2006).

5. Wouters, M. & Carusotto, I. Excitations in a Nonequilibrium Bose-Einstein Condensate of Exciton Polaritons. *Phys. Rev. Lett.* **99,** 140402 (2007).

6. Ivchenko, E. L. *Optical spectroscopy of semiconductor nanostructures /*. (Alpha Science,, c2005.).

7. Walck, S. N. & Reinecke, T. L. Exciton diamagnetic shift in semiconductor nanostructures. *Phys. Rev. B* **57,** 9088–9096 (1998).

8. Nikolaev, V. V. & Portnoi, M. E. Theory of the excitonic Mott transition in quasi-two-dimensional systems. *Superlattices and Microstructures* **43,** 460–464 (2008).

9. Strobel, R., Eccleston, R., Kuhl, J. & Köhler, K. Measurement of the exciton-formation time and the electron- and hole-tunneling times in a double-quantum-well structure. Phys. Rev. B 43, 12564–12570 (1991).

10. Kumar, R., Vengurlekar, A. S., Prabhu, S. S., Shah, J. & Pfeiffer, L. N. Picosecond time evolution of free electron-hole pairs into excitons in GaAs quantum wells. Phys. Rev. B 54, 4891–4897 (1996).

11. J. Kasprzak, R. André, Le Si Dang, I. A. Shelykh, A. V. Kavokin, Yuri G. Rubo, K. V. Kavokin, and G. Malpuech, Build up and pinning of linear polarization in the Bose condensates of exciton polaritons, *Phys. Rev.* B **75**, 045326 (2007).

12. Amthor, Matthias, et al. "Electro-optical switching between polariton and cavity lasing in an InGaAs quantum well microcavity." *Optics express* **22.25**, 31146 (2014).
